# Advances in Therapeutic Approaches to Extend Healthspan: a perspective from the 2^nd^ Scripps Symposium on the Biology of Aging

**DOI:** 10.1111/acel.12620

**Published:** 2017-06-06

**Authors:** Paul D. Robbins, Laura J. Niedernhofer

**Affiliations:** ^1^ Department of Molecular Medicine and the Center on Aging The Scripps Research Institute Jupiter FL 33458 USA

## Abstract

The 2^nd^ Scripps Florida Symposium on The Biology of Aging entitled ‘Advances in Therapeutic Approaches to Extend Healthspan’ was held on January 22^nd^–25^th^, 2017 at The Scripps Research Institute in Jupiter, Florida. The meeting highlighted a variety of therapeutic approaches in animal models of aging that either are or soon will be in clinic trials. For example, drugs targeting senescent cells, metformin, rapalogs, NAD precursors, young plasma, mitochondrial‐targeted free radical scavengers, stem cells, and stem cell factors all have shown significant preclinical efficacy. This perspective, based on presentations and discussions at the symposium, outlines the current and future state of development of therapeutic approaches to extend human healthspan.

The 2^nd^ Scripps Florida Symposium on The Biology of Aging entitled ‘Advances in Therapeutic Approaches to Extend Healthspan’ was held on January 22^nd^–25^th^, 2017 at The Scripps Research Institute in Jupiter, Florida. The goal of the symposium was to bring together leaders in the fields of aging and drug development to discuss strategies for identifying and developing therapeutic approaches to extend human healthspan. This symposium made it highly evident that the biology of aging field is moving quickly toward translational research. At the symposium, there were numerous reports of successful drug screens and drug testing in a variety of model systems. There was also an overall sense of excitement, given that multiple therapeutic modalities, including young plasma, recombinant proteins, and small molecules, extend healthspan and lifespan in model organisms and that clinical trials to test the efficacy of these treatment modalities on healthspan and resilience have been initiated.

The concept of Geroscience, defined as the understanding of the relationship between aging and age‐related diseases and preventing/delaying disease by targeting fundamental mechanisms of aging (Kennedy *et al*., 2014), was an underlying theme of the symposium. As reiterated by the keynote speakers, Jay Olshansky, Felipe Sierra and Steve Austad, aging is the main risk factor for most chronic diseases. Thus, developing approaches to therapeutically target aging should be a funding priority for the majority of institutes at the National Institutes of Health, as well as other funding agencies, philanthropists, and foundations (Kaeberlein *et al*., 2015). The socioeconomic need to extend human healthspan also was made clear. As a consequence of the advances in prevention and treatment of infectious diseases, there will be an unprecedented increase in the number of persons over 65 over the next decades. By 2035, the cost of treating Americans 65 years and older is expected to be over $2 trillion annually. Thus, finding ways to prevent all age‐related diseases is one of the most imperative biomedical pursuits.

A common theme arising from the symposium was the need for appropriate model organisms to study aging and age‐related disease including models carrying reporters of senescence, mitochondrial function, autophagy and reactive oxygen species (ROS). The use of these reporters or testing of therapeutics needs to be performed in aged model organisms, a problem that has also plagued the cancer field because of the cost and time involved, at least in rodent models. Here, the National Institute on Aging (NIA) Interventions Testing Program in mice (ITP) and in *Caenorhabditis elegans* (*C. elegans)* (CITP) have made significant contributions to the identification of drugs/compounds able to extend lifespan (Nadon *et al*., 2008; Strong *et al*., 2008; Harrison *et al*., 2009, 2014; Miller *et al*., 2011). For example, aspirin, rapamycin, acarbose, 17α‐estradiol, Protandim and nordihydroguaiaretic acid (NDGA) extend the lifespan of at least one sex of genetically heterogeneous mice. With this number of current ‘hits’ and likely future ‘hits’, it is possible that shared mechanism(s) of action will emerge, illuminating novel biology regarding the pathways that drives aging. Unfortunately, the ITP is not incorporating reporters into their heterogeneous mouse strains. The development of novel model organisms of aging, including the African Killifish (*Nothobranchius furzeri*) (Harel *et al*., 2015; Valenzano *et al*., 2015) and different strains of nematodes that can be easily modified to carrying specific reporters should help not only identify pathways of aging, but also in the screening for therapeutics.

Despite the ITP, CITP, and new models of aging, there still is a need for an expansion of efforts to measure the effects of drugs/compounds on healthspan or resilience, which has greater translational relevance (Niedernhofer *et al*., 2017). Thus, many investigators are beginning to incorporate functional analysis of aged mice undergoing therapeutic interventions, for example, echocardiography (Chiao & Rabinovitch, 2015). Also, mouse models of accelerated aging afford rapid testing of interventions and measurement of the impact on age‐related symptoms and functional decline. These models include those of Hutchinson–Guilford Progeria Syndrome (HGPS) and XFE progeroid syndrome, which mimic many aspects of natural aging, including senescence, stem cell dysfunction, mitochondrial dysfunction, metabolic changes, and predisposition to common age‐related diseases (Harkema *et al*., 2016). Murine strains with tissue‐specific accelerated aging also have been developed to aid in the discovery of which organ(s) are sentinel for driving organismal aging. Preliminary data point toward aging of the immune system as being critical for systemic aging (Laura Niedernhofer). Consistent with this is the key role of hemocytes in tissue repair of invertebrates (Neves *et al*., 2016) and the observation that transplantation of healthy, young immune cells into mice after total body irradiation reduced the burden of senescent cells (Andrei Gudkov).

mTOR continues to be a favored molecular target for aging, given the ability of rapamycin and rapalogs to extend lifespan in multiple model systems (Harrison *et al*., 2009; Liao *et al*., 2016). Remarkably, there is still no consensus on its mechanism of action. Upstream of mTOR, an age‐related decline in proteolysis by the lysosome may limit circulating amino acids, driving mTOR activation (Wolfson *et al*., 2017). Downstream of mTOR, 4EBP1 is a critical effector that regulates protein translation. 4EBP1 appears to be regulated by inflammation (a key feature of aging) in a sex‐specific manner in mice (Brian Kennedy). However, much work remains to define the key age‐related activator of mTOR and downstream effector(s).

Biologics represent another exciting therapeutic approach to improve healthspan. Heterochronic parabiosis or repeated injection of young plasma into old mice results in improvement in the function of some tissues, including the central nervous system (Conboy *et al*., 2005, 2013; Conboy & Rando, 2012; Loffredo *et al*., 2013; Villeda *et al*., 2014; Smith *et al*., 2015). This suggests that serum factors can be identified that slow or even reverse certain aspects of aging. This is true not only for naturally aged mice, but also for murine models of progeria such as *Zmpste24* ^−/−^ and *Ercc1* ^−/∆^ mice where parabiosis with young wild‐type mice improved some aging phenotypes (Johnny Huard). There have been several factors identified in the serum of young animals that have been proposed to act as antigeronic factors, including oxytocin, TIMP2, mesencephalic astrocyte‐derived neurotrophic factor (MANF) and growth differentiation factor 11 (GDF‐11) (Loffredo *et al*., 2013; Sinha *et al*., 2014; Neves *et al*., 2016). GDF‐11 has offered a potent reminder that accurate detection of serum proteins is tremendously difficult due to interference by isoforms, receptors, multiple activity states (pre‐, pro‐ forms) and serum binding partners (Walker *et al*., 2016). Levels of other proteins, such as GM‐CSF and TIMP‐2, also appear change significantly with aging (Bonnema *et al*., 2007; Kim *et al*., 2011). Remarkably, injection of mice with human fetal cord blood significantly improved the cognitive function of mice. This was mimicked by injecting recombinant TIMP‐2 whereas, in contrast, depletion of TIMP‐2 from fetal cord blood abrogated its positive effects on cognition (Tony Wyss‐Coray). These observations demonstrate that tissue rejuvenation and improved cognitive function could be attainable by treatment of the elderly with young plasma and ultimately recombinant proteins. Clearly, more effort needs to be placed on identifying and understanding how young serum slows aging. Results from an ongoing clinical trial to assess the effect of young plasma on Alzheimer's disease will provide significant impetus to identify antigeronic serum factors (Middeldorp *et al*., 2016).

An emerging paradigm in the field of aging is that the burden of senescent cells increases with age in multiple tissues and reducing this senescent cell burden improves healthspan. The development and characterization of two transgenic mouse models, p16‐INK‐ATTAC and p16‐3MR mice, clearly demonstrated that senescent cells play a role in driving aging and age‐related diseases (Baker *et al*., 2011, 2016; Childs *et al*., 2016). The reduction of senescent cells in the p16‐3MR mice reduced atherosclerosis, improved metabolism, prevented tumor metastasis and reduced osteoarthritis in an injury model (Demaria *et al*., 2017). Thus, the hunt is on for senolytics or drugs that specifically kill senescent cells (Zhu *et al*., 2015, 2016, 2017; Chang *et al*., 2016; Wang *et al*., 2016). Screening of compounds in senescent human and mouse cultures revealed a number of classes of drugs/compounds that can induce apoptosis of senescent cells. These senolytics include the tyrosine kinase inhibitor Dasatinib (D), the flavonoid Quercetin (Q), the Bcl‐2 family inhibitor Navitoclax, piperlongumine and Hsp90 inhibitors (Zhu *et al*., 2015; Chang *et al*., 2016; Wang *et al*., 2016; Zhu *et al*., 2016; Schafer *et al*., 2017; Zhu *et al*., 2017; Paul Robbins). Treatment of a mouse model of accelerated aging with D+Q or the Hsp90 inhibitor 17‐DMAG extended their healthspan (Zhu *et al*., 2015). Similarly, treatment of naturally aged mice with D+Q improved heart function and improved metabolism, especially in mice on a high‐fat diet (Roos *et al*., 2016). Whether these or more optimized senolytics will have similar positive effects on human healthspan is still unclear, but clinical trials are being planned to determine their effectiveness.

It is important to note that it is likely that no one senolytic will be effective in eliminating all types of senescent cells. Individual senolytic compounds are apt to have tissue‐specific and even cell type‐specific effects. Furthermore, there is increasing evidence that different drivers of senescence can lead to differences in how senescence manifests, which in turn could have a variable impact on the senescent cell's environment. Another complexity is that some drugs/compounds affect expression of senescent‐associated secretory phenotype (SASP) factors without eliminating the senescent cells (Tilstra *et al*., 2012; Laberge *et al*., 2015; Xu *et al*., 2015). For example, rapamycin and NF‐κB and JAK inhibitors reduce SASP and other markers of senescence without actually inducing cell death. This class of compounds, termed senomorphics, also may affect healthspan through reducing the cell nonautonomous effects of senescence on aging. Clearly documenting whether a compound functions as a senolytic or senomorphic *in vivo* will require the development of new reporters and methodologies.

Adult stem cell function is known to decline with aging. However, it has taken longer to demonstrate that the loss of stem cell function contributes to aging and is not simply a consequence of it. Treatment of a mouse model of accelerated aging with two types of young stem cells, muscle derived stem/progenitor cells (MDSPCs) and bone marrow‐derived mesenchymal stem cells (MSCs) extends healthspan and lifespan (Lavasani *et al*., 2012). This was also recapitulated in aged rats using adipose‐derived mesenchymal stem cells (Kim *et al*., 2015). Although the exact mechanism for how these stem cell populations affect aging is unknown, preliminary data suggest that their effect is mediated by factors secreted by young, but not old stem cells. These factors appear to reduce cellular senescence and improve the function of endogenous, aged stem cells. Whether these stem cell‐derived soluble factors are the same as those found in young plasma is currently unknown. In addition to loss of stem cell function with age, there is also evidence that progenitor cell populations in muscle contribute to driving aging (Johnny Huard). Importantly, this age‐related stem cell dysfunction appears reversible. New data was presented pointing toward the existence of cardiac stem cells necessary for tissue regeneration (Nadal‐Ginard *et al*., 2014; Smith *et al*., 2014). Currently, senotherapeutics are being tested on these cardiac stem cells to determine whether senescence is an underlying factor in cardiac aging. This will undoubtedly remain an area of intense research spanning from continued investigations into fundamental mechanisms of aging to clinical trials.

The occurrence of genetic variants in humans that affect longevity and health in old age offers an ideal starting point for the systematic identification of targets for interventions that affect healthspan. Examples of these ‘natural mutants’ are human centenarians and super‐centenarians where rare variants in the coding sequence of IGF‐1R, FOXO3a, and SIRT6 have been identified (Suh *et al*., 2008). An alternative approach to identifying gene variants important for healthy aging is to link a human phenotype, such as presence or absence of age‐related disease, to a genotype. The extensive exome sequencing being performed by companies such as 23andMe and Regeneron will facilitate the discovery of genes linked to extended healthspan defined as the avoidance of age‐related disease (Dewey *et al*., 2016). However, many rare variants lie in noncoding regions or transcriptional regulatory regions located at significant distances from their effector gene, making their identification and characterization of their mechanisms of action quite difficult.

Clear evidence that the field of aging is moving forward quickly is the number of ongoing or soon‐to‐be‐initiated clinical trials. Importantly, the use of specific short‐term clinical endpoints to determine if resilience or function of a specific tissue could be improved is employed to reduce study size, duration, and cost. For example, short‐term treatment of a cohort of elderly people with a rapamycin analogue (rapalog) was tested for its ability to improve immune function (Mannick *et al*., 2014). The inclusion of additional endpoints provides further information about not only the effect of the intervention on immune function, but also on other aspects of aging that might be modulated and measured in future clinical trials. Similarly, short‐term clinical trials with the mitochondrial‐targeted SS‐31 peptide are in progress for heart disease based on very promising preclinical data (Dai *et al*., 2011; Siegel *et al*., 2013). In contrast, a well‐controlled clinical study to examine the effect of high dose resveratrol on diabetes showed no positive effect, despite promising preclinical data (Jill Crandell). Unfortunately, a negative result might only mean that the wrong dose or dosing regimen was used or the wrong patient population enrolled.

These short‐term treatment trials with well‐defined, disease‐specific endpoints are in contrast to the highly anticipated Treating Aging with Metformin (TAME) trial. The TAME trial is designed to enable evaluating whether metformin extends the healthspan of humans albeit in a rapid 3‐5 year format (Barzilai *et al*., 2016; Newman *et al*., 2016). It is hoped that the TAME trial will serve as a template for pharmaceutical companies to do future testing of drugs aimed at targeting fundamental mechanisms of aging.

In addition to the exceptional science presented, several important issues were raised during the panel discussion. One of these issues is that it is impossible to stop the world's population from getting older, but it is possible to make this older population healthier. A second issue is emphasizing that the push is for therapies that ablate age‐related disease, not extend lifespan. A small lifespan extension may be a ‘side effect’ of this therapeutic approach. A third issue is that we already have therapeutics that extend health like statins and insulin, but patient compliance is not perfect. How compliant will people be with taking additional preventative therapies? Finally, dialogue about how society should adapt, both socially and economically, to a healthier older population is needed.

The Glenn Foundation and the American Federation for Aging Research (AFAR) are doing an admirable job in funding top quality aging research, but with limited budgets. However, it also is clear that support from the private sector will be essential for moving clinical trials forward as there is a huge need for funding from sources other than the NIH to expedite aging research. The successful completion of the first clinical trial demonstrating that human healthspan can be extended is anticipated to instigate tremendous interest in the field by biotech investors and potentially philanthropists. Thus, this first proof‐of‐principle clinical trial and funding support for it is considered a significant hurdle that must be crossed to accelerate funding and progress in the field.

Another hurdle is the lack of biomarkers for assessing the efficacy of a compound in slowing aging, which can be translated from animal models to human trials. A defined biomarker of biological aging would facilitate not only predicting who should be enrolled in clinical trials for aging therapeutics, but also in determining the efficacy of a treatment regimen. A third hurdle is the need for infrastructure for running large‐scale, aging clinical trials, analogous to NCI Comprehensive Cancer Centers. Here, leveraging and expanding the existing infrastructure within the NIH‐funded Clinical and Translational Science Award (CTSA) program, Claude D. Pepper Older Americans Independence Centers (OAIC), Nathan Shock Centers of Excellence and Glenn Centers for Research in Aging could help facilitate successful clinical trials. There also is a lack of geriatricians with appropriate training in clinical trial design. This combination of lack of biomarkers, geriatricians experienced in clinical trials, and a successful trial template creates a unique challenge that will be difficult, but critical to overcome. Despite these barriers to the field, all of which can be overcome with research funding and appropriate planning, there likely will be a number of significant preclinical and clinical advances by the 3^rd^ Scripps Symposium on the Biology of Aging in January of 2019.

## Funding

The authors also would like to thank the sponsors of the Scripps Florida Symposium including Elizabeth Fago, Nuvista Institute for Healthy Living, Joseph A. Pesenti & Bernice Fain Pesenti Foundation, Novartis, Florida Power and Light, Jupiter Medical Center, American Federation of Aging Research, Chip Block, and Leanna Landsman. P.R. and L.J.N are supported by Program Project Grant AG043376 from the National Institutes of Health.

## Conflict of interest

None declared.
